# Lack of Biological Plausibility and Major Methodological Issues Cast Doubt on the Association between Aspartame and Autism. Comment on Fowler et al. Daily Early-Life Exposures to Diet Soda and Aspartame Are Associated with Autism in Males: A Case-Control Study. *Nutrients* 2023, *15*, 3772

**DOI:** 10.3390/nu16050675

**Published:** 2024-02-28

**Authors:** Sabrina Ayoub-Charette, Tauseef Ahmad Khan, Laura Chiavaroli, Bernadene A. Magnuson, John L. Sievenpiper

**Affiliations:** 1 Department of Nutritional Sciences, Temerty Faculty of Medicine, University of Toronto, Toronto, ON M5S 1A8, Canada; sabrina.ayoubcharette@mail.utoronto.ca (S.A.-C.); tauseef.khan@utoronto.ca (T.A.K.); laura.chiavaroli@alumni.utoronto.ca (L.C.); 2 Toronto 3D Knowledge Synthesis and Clinical Trials Unit, Clinical Nutrition and Risk Factor Modification Centre, St. Michael’s Hospital, Toronto, ON M5C 2T2, Canada; 3 Li Ka Shing Knowledge Institute, St. Michael’s Hospital, Toronto, ON M5B 1T8, Canada; 4 Health Science Consultants Inc., Collingwood, ON L9Y 0Z5, Canada; berna@bernamagnuson.com; 5 Division of Endocrinology and Metabolism, Department of Medicine, St. Michael’s Hospital, Toronto, ON M5C 2T2, Canada; 6 Department of Medicine, Temerty Faculty of Medicine, University of Toronto, Toronto, ON M5S 1A8, Canada

The case–control study by Fowler et al. [1] assessed the association between pregnant and breastfeeding women’s consumption of diet soda/beverage and aspartame, and the incidence of autism spectrum disorder in their children. The conclusions appear unjustified and unreliable, based on a lack of plausible biological mechanism and methodological issues, which put the data at a high risk of reverse causality.

Mothers were asked only to recall the frequency of their intake of diet soda/beverage and of low-calorie sweetener packets, including their intake during pregnancies that occurred up to 30 years earlier, which is highly unreliable. No other dietary information was collected, despite associations of other dietary factors with autism spectrum disorder [2].

In humans, aspartame is completely hydrolyzed by digestive enzymes into phenylalanine (50%), aspartate (40%), and methanol (10%) [3]. Intact aspartame does not enter the blood, nor the large intestine. Aspartame therefore is never present in the placenta, amniotic fluid, or breast milk [4]. The authors imply the transplacental passage of aspartame in humans based on a recent study [5] that explicitly stated they did not measure aspartame “due to its rapid degradation … upon ingestion”.

The authors discuss the mechanisms of the metabolites of aspartame implicated in the development of autism spectrum disorder, based primarily on high-dose animal studies. However, for even the highest human consumers of aspartame, these mechanisms are not biologically plausible. The contribution of amino acids from aspartame is trivial in comparison to dietary proteins and results in no change in human blood levels nor any change in brain amino acid uptake [3]. Using dietary surveys, when all sources of methanol are considered (including methanol present in or produced during digestion of fruits, vegetables, juices, coffee, alcoholic beverages, and other food additives, plus the endogenous methanol production), the methanol from aspartame in humans ranged from 1 to 10% of total methanol [6].

Another significant limitation of this study is the high risk of reverse causality, where high cardiometabolic risk—encompassing obesity, type 2 diabetes, and cardiovascular disease—prompts the increased intake of low-calorie sweeteners as a risk mitigation strategy. Reverse causality is well documented in the literature evaluating low-calorie sweeteners [7,8]. The authors failed to account for or adjust for key cardiometabolic risk factors, such as maternal obesity and diabetes, which are established risk factors for autism spectrum disorder. Although the authors recognize these limitations, such shortcomings substantially undermine the study’s validity.

In summary, the association between diet soda/beverage and aspartame exposure and autism spectrum disorder reported by Fowler et al. is unreliable, not biologically plausible and is confounded by reverse causality. Caution should be exercised when suggesting that a mothers’ dietary habits play a possible role during pregnancy based on unreliable associations. Therefore, these findings should be interpreted with significant caution, and we urge for higher standards of quality in nutritional epidemiological studies.
